# Lung Transplantation With Elevated Pulmonary Vascular Resistance: Insights From the United Network for Organ Sharing Database

**DOI:** 10.1016/j.atssr.2025.01.022

**Published:** 2025-02-25

**Authors:** Anand Maligireddy, Ahmad Jabri, Chaitanya Rojulpote, Laith Alhuneafat, Herbert Aronow, Jonathan Haft, Pedro Villablanca, Rana Awdish, Bryan Kelly, Domingo Franco-Palacios, Farhan Nasser, Gillian Grafton, Hassan Nemeh, Kyle Miletic, Lisa Allenspach, Vikas Aggarwal

**Affiliations:** 1 The Wright Center for Graduate Medical Education, Scranton, Pennsylvania; 2 Division of Cardiovascular Medicine, Department of Internal Medicine, Henry Ford Hospital, Detroit, Michigan; 3 Division of Cardiology, Department of Medicine, St Louis University Hospital, St Louis, Missouri; 4 Division of Cardiovascular Medicine, University of Minnesota, Minneapolis, Minnesota; 5 Michigan State University College of Human Medicine, East Lansing, Michigan; 6 Department of Cardiothoracic Surgery, University of Michigan, Ann Arbor, Michigan; 7 Division of Pulmonary Medicine, Department of Internal Medicine, Henry Ford Hospital, Detroit, Michigan; 8 Department of Osteopathic Medical Specialties, Michigan State University College of Osteopathic Medicine, East Lansing, Michigan; 9 Division of Cardiovascular Medicine, The Metro Health System/Case Western Reserve University, Cleveland, Ohio; 10 Division of Cardiothoracic Surgery, Department of Surgery, Henry Ford Hospital, Detroit, Michigan

## Abstract

**Background:**

Pulmonary hypertension is a significant challenge in patients requiring a lung transplant, often being manifested with severe complications such as high pulmonary vascular resistance (PVR). Although medical treatments have extended median survival, pulmonary hypertension remains a progressive and life-threatening condition. Lung transplantation offers potential for improved outcomes, supported by advancements in surgical techniques, donor lung preservation, immunosuppression, and posttransplantation care.

**Methods:**

Using the United Network for Organ Sharing database, we analyzed adult patients undergoing double lung transplantation from October 1, 2002, to September 30, 2022. Our focus was on patients with elevated PVR (≥6 Wood units), with or without underlying lung parenchymal disease. Trends in transplantation, survival rates, and impact of center volume on outcomes were examined.

**Results:**

Of 24,921 double lung transplant recipients, 2755 patients had PVR ≥6 Wood units. There was a significant upward trend in annual procedures, with increased use of extracorporeal support during surgery. Higher volume centers (performing >33 transplants annually) demonstrated better survival rates. Elevated PVR was independently associated with higher mortality, highlighting its importance in patient selection and management.

**Conclusions:**

Lung transplantation remains a critical option for patients with end-stage lung disease, including those with high PVR. Improved outcomes at high-volume centers underscore the importance of institutional experience and expertise.

**In Short**

▪ During the past 2 decades, advancements in lung transplantation, including improved surgical techniques and perioperative management, have enhanced outcomes, particularly for patients with high pulmonary vascular resistance.
▪ High-volume lung transplant centers are associated with better survival rates, emphasizing the importance of institutional experience for managing complex cases.
▪ The use of extracorporeal membrane oxygenation as a bridge to lung transplantation has increased significantly, highlighting its critical role in managing high-risk pulmonary hypertension patients.

During the past 20 years, basic and translational science discoveries have advanced our understanding of the underlying pathobiologic mechanisms causing pulmonary arteriopathy, leading to the development of several effective treatment options and overall better outcomes for patients with pulmonary hypertension (PH). With more effective therapies, median survival from the time of diagnosis for a newly diagnosed patient with PH has increased to 7 years, depending on the cause of PH, response to treatment, and other patient factors.^1^ Nevertheless, PH remains a life-threatening and life-limiting disease for most patients.^2^ For such patients who are refractory to or progress despite medical treatment of PH, lung transplantation remains the only viable option for improving survival.

During the past 2 decades, parallel advancements in the field of lung transplantation have yielded improved outcomes, including the establishment of revised guidelines for selecting candidates, enhanced surgical techniques, improved methods for donor lung preservation, advances in suppressing and treating allograft rejection, and development of prophylaxis protocols to decrease the risk of opportunistic infection.^3^ An increasing number of patients undergoing evaluation for lung transplantation are extremely ill and require ventilator and extracorporeal life support as a bridge to transplantation.^4^ Early posttransplantation survival has improved with the implementation of advances in surgical techniques and perioperative management. Early complications, such as surgical complications or primary graft dysfunction, continue to threaten lung allograft function and viability and significantly affect recipient survival and long-term outcomes. This is particularly true for higher risk subsets, such as those with PH undergoing lung transplantation.

The current literature does not comprehensively reflect the contemporary use and efficacy of lung transplantation as the last treatment option for patients with severe pulmonary arteriopathy. This study uses the United Network for Organ Sharing database to examine the outcomes of double lung transplant, focusing on patients with high pulmonary vascular resistance (PVR) and overall double lung transplant recipients. We investigate mortality rates across transplant centers of varying volumes, particularly for high-PVR patients, and 20-year trends in double lung transplant use, including procedure numbers, extracorporeal membrane oxygenation (ECMO) use, and waitlist times.

## Patients and Methods

### Study Population

This study uses data from the United Network for Organ Sharing service portal. All patients 18 years and older who underwent lung transplantation between October 1, 2002, and September 30, 2022, were included. Given current recommendations for double lung transplant in patients with PH, those with single lung transplant were excluded. Patients with any prior solid organ transplant and those who underwent multiorgan transplants were also excluded (Supplemental Figure). This study was conducted with deidentified patient information and was therefore deemed exempt from institutional review board approval.

### Center Volume

**Figure 1:**
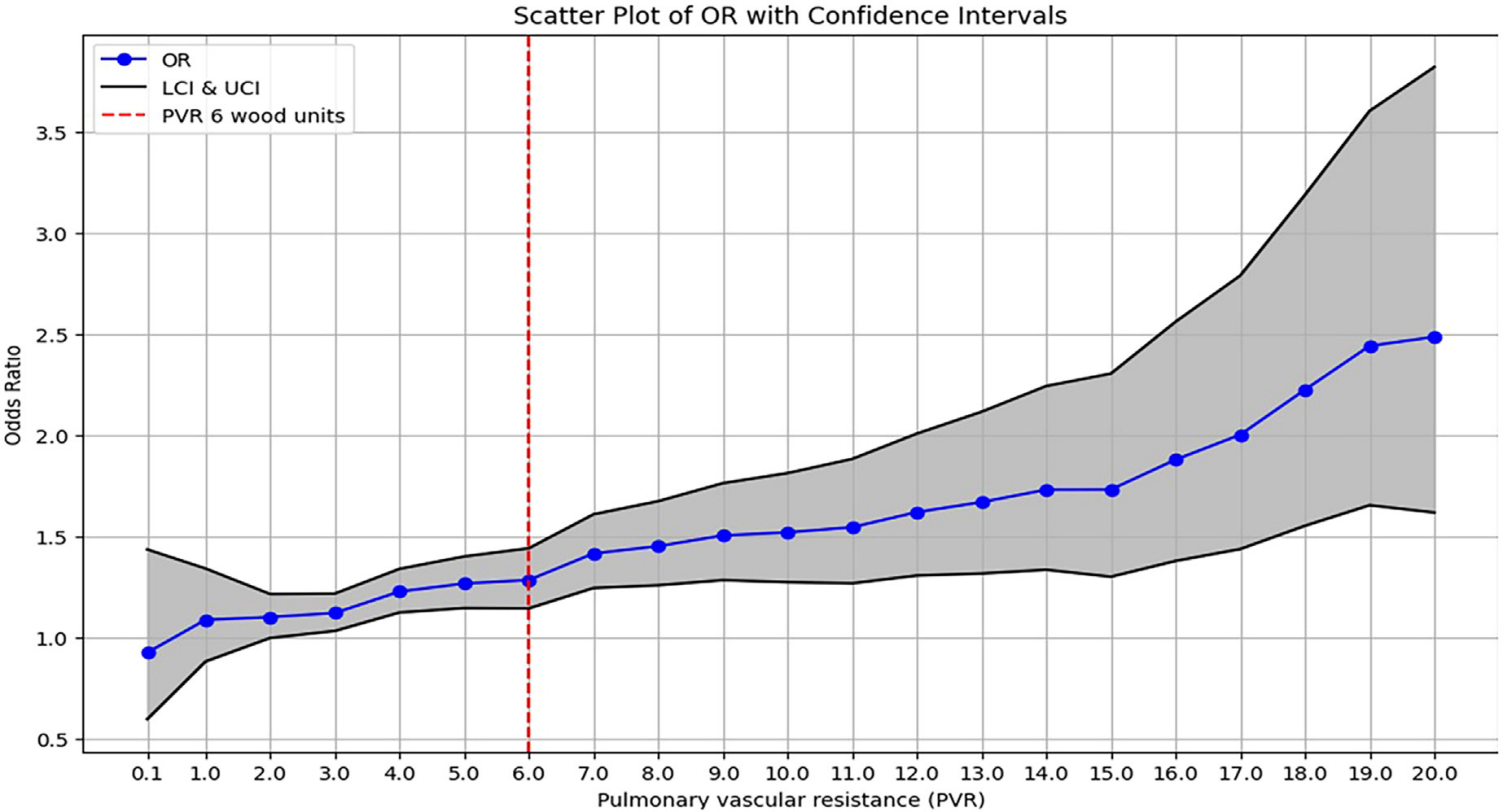
Scatter plot demonstrating a relationship between pulmonary vascular resistance (PVR) and posttransplant mortality within 1 year. (LCI, lower confidence interval, OR, odds ratio; UCI, upper confidence interval.)

Centers were stratified according to whether they performed ≥33 (higher volume) or <33 (lower volume) lung transplants per year based on a study by Yang and coworkers,^5^ in which a threshold of 33 annual lung transplants was associated with 1-year survival. PVR was related to mortality at 1 year by plotting the odds ratio for mortality against PVR in Wood units. Prestudy analysis revealed a critical PVR threshold of >6 Wood units, for which the odds ratio for mortality significantly increased (Figure 1). This inflection point was both visually apparent and statistically significant. Based on this finding, we defined high PVR as ≥6 Wood units for comparing outcomes between low- and high-volume centers.

### Statistical Methods

Frequencies and percentages were used to describe categorical variables and medians with interquartile ranges for continuous variables. Mantel-Haenszel *χ*^2^ and Wilcoxon rank sum (Mann-Whitney) testing were employed for respective unadjusted analyses. Survival analysis was conducted with unadjusted and adjusted Cox regression for comparisons. *P* values < .05 were considered statistically significant.

## Results

### Baseline Characteristics

Of 24,921 patients who received double lung transplants, a subset of 2755 patients demonstrated elevated PVR ≥6 Wood units (high-PVR group). This subgroup's demographic profile starkly contrasts with the broader cohort, particularly in gender composition, female participants representing 54.6% in the higher PVR group, significantly higher than the 42% in the overall transplant cohort (*P* < .05). Ethnic diversity also varied markedly; 67% of the patients in the higher PVR subgroup were White (White race represented 79% of all patients receiving transplants). Conversely, of minority patients, African Americans (17.9% vs 10.1%) and Hispanic/Latino patients (10.3% vs 8%) had a higher rate of PVR ≥6 Wood units (*P* < .01).

Age profiles between the groups were more aligned, with median ages of 57 years in the overall cohort and 56 years in the high-PVR group (*P* < .05). The prevalence of specific medical conditions also diverges. Notably, the high-PVR group showed a more frequent history of cerebrovascular disease (3.1% as opposed to 0.8% in the general cohort; *P* < .05). In comparison, malignant disease was less common (6.2% vs 7.6%; *P* = .002).

The percentage of patients requiring mechanical ventilation at listing was lower in the high-PVR group compared with others (2.4% vs 3.3%; *P* < .05); but during the transplantation admission, the occurrence was higher in the high-PVR group (6.1% vs 7%; *P* < .05). ECMO use, meanwhile, did not significantly differ at listing (2.5% in the high-PVR group vs 2.7%; *P* = 0.425) but was higher during the admission requiring transplantation (7.5% vs 5.8%; *P* < .05).

The high-PVR group also exhibited a greater need for intensive care, with 20.1% in the intensive care unit and 13.2% hospitalized but not in the intensive care unit, compared with 13.9% and 10%, respectively, in the overall group (*P* < .01), hinting at a more complex or severe disease state in these patients. In addition, lung function test results displayed significant contrasts; the high-PVR group's median predicted forced expiratory volume in 1 second was 56% against 46% in the overall group, and their forced vital capacity was 51% compared with 34% (both *P* < .01). Their median oxygen requirement was also marginally higher at 4 L/min vs 3 L/min in the overall cohort (*P* < .01).

Of the primary diagnoses leading to transplantation, between the overall cohort and those with PVR ≥6 Wood units, idiopathic pulmonary fibrosis emerged as the most common diagnosis in both groups. It was slightly more prevalent in the overall cohort (29.5%) than in the high-PVR group (27.5%). Notably, pulmonary arterial hypertension (PAH) was significantly associated with a higher PVR and accounted for 20.5% of the cases, underscoring its strong association. At the same time, it was less prominent in the overall cohort. Sarcoidosis and secondary pulmonary fibrosis also showed higher occurrence rates in the high-PVR group (Supplemental Table 1).

### Center Volume and Mortality

In patients with PVR ≥6 Wood units, compared with centers with higher volumes, those with lower volumes exhibited a greater hazard for 1-year mortality (15.2% vs 13.5%; hazard ratio, 1.39; 95% CI, 1.13-1.71; *P* < .01). A similar pattern persisted for cases with a PVR ≥2, for which lower volume centers again presented a higher hazard ratio of 1.19 and an incidence rate of 13.41%. In contrast, for the more narrowly defined patient groups, such as those with PVR ≥6 and forced vital capacity either below 70% or above 70%, the hazard ratios at low-volume centers rose to 1.27 (95% CI, 0.98-1.64) and 1.88 (95% CI, 1.25-2.83), respectively, with *P* value > .05 (Table).

**Table:** Risk-Adjusted Mortality Rate

Variable	Low-Volume Center (<33 total), HR	High-Volume Center (≥33 total)	Low-Volume Center (<33 total)	High-Volume Center (≥33 total)
Lung transplant with PVR ≥2 Wood units	1.19 (1.08-1.30; *P* < .05)	1.0 (reference)	1054/7860 (13.41%)	942/8608 (10.94%)
Double lung transplant with PVR ≥6 Wood units	1.39 (1.13-1.71; *P* < .05)	1.0 (reference)	211/1391 (15.17%)	184/1364 (13.49%)
Double lung transplant with PVR ≥6 Wood units and FVC ≥70% predicted	1.88 (1.25-2.83; *P* < .05)	1.0 (reference)	71/411 (17.27%)	64/433 (14.78%)

FVC, forced vital capacity; HR, hazard ratio; PVR, pulmonary vascular resistance.

### Causes of Death

As for the causes of death 1 year after transplantation, bacterial septicemia was the leading cause in both cohorts but was more prevalent in the high-PVR group (12.6% vs 10.7%). The incidences of multiple organ failure (8.9% in both) and respiratory failure (9.2% vs 7.3%) were significant in both, whereas primary graft failure (5.5% vs 6.2%) and cardiac arrest (6.5% vs 5.0%) were also common causes of death.

### One-Year Mortality by Respiratory Failure Cause

A focused analysis of 1-year mortality stratified by the top causes of respiratory failure revealed variations between the high-PVR and low-PVR groups. Patients with chronic obstructive pulmonary disease and idiopathic pulmonary fibrosis demonstrated higher 1-year mortality rates in the high-PVR group compared with the low-PVR group. Notably, the total 1-year mortality rate was higher in the high-PVR group (15%) compared with the low-PVR group (12%), emphasizing the additional risks posed by elevated PVR. Specific findings are summarized in Supplemental Table 2.

### Contemporary Trends in Use of Lung Transplantation for PAH

**Figure 2:**
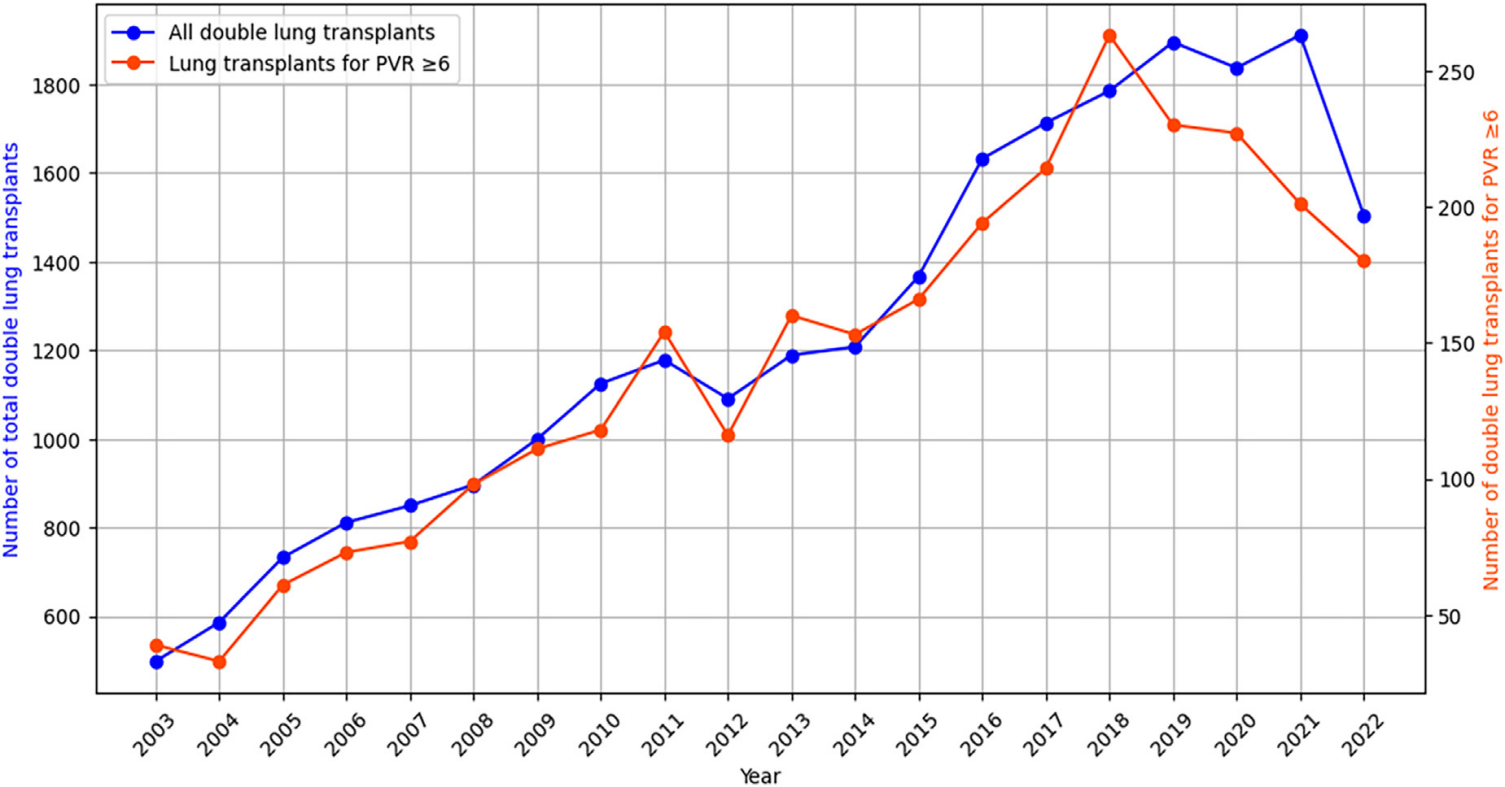
Temporal trends showing the annual number of total lung transplants and lung transplants in individuals with pulmonary vascular resistance (PVR) ≥6 Wood units.

During the past 2 decades, there has been a significant increase in the annual total of double lung transplants, encompassing cases in which patients have PVR ≥6 Wood units. Since 2003, the trend for double lung transplant has consistently risen, starting from 498 transplants in 2003 and peaking at 1910 transplants in 2021. Specifically, transplants for patients with a PVR ≥6 Wood units have also seen a peak, reaching 263 transplants in 2018, up from 39 transplants in 2003 (Figure 2).

**Figure 3:**
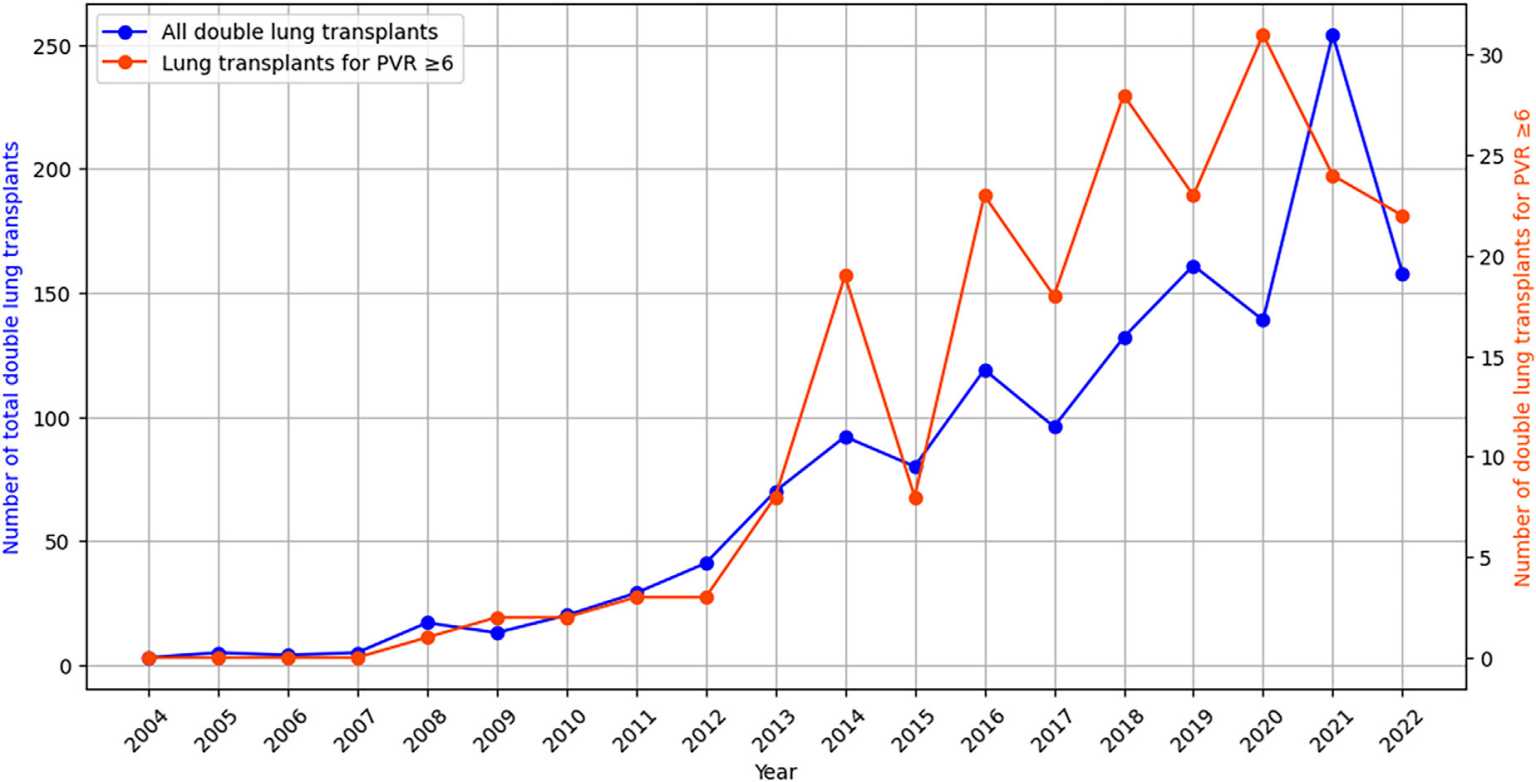
Temporal trends showing extracorporeal membrane oxygenation use in all lung transplants and lung transplants for pulmonary vascular resistance (PVR) ≥6 Wood units.

During the study period, the use of ECMO in double lung transplants began at 3 in 2004, demonstrating a significant increase over the years to reach a peak of 254 transplants in 2021. In the high-PVR group, there was no recorded use of ECMO until 2008, which marks the beginning of its application in these more complex cases. From that point, the number of high-PVR cases gradually increased from a single case in 2008 to a peak of 31 cases in 2020. This subset of data highlights the specific trends in ECMO use for patients with high PVR, showcasing its increasing role in managing complex lung transplant cases (Figure 3).

**Figure 4:**
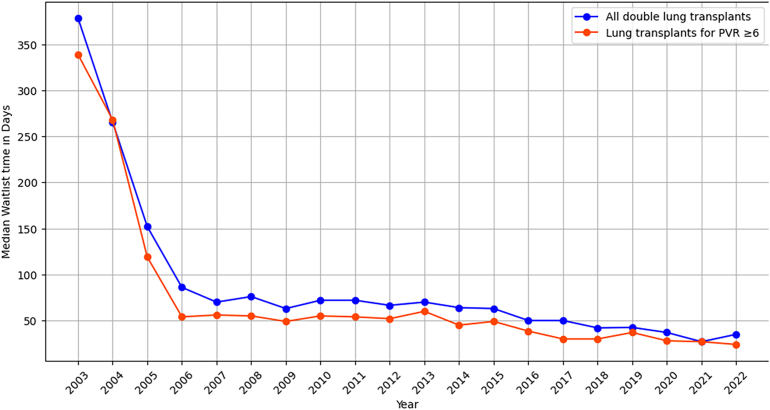
Temporal trends in waitlist times before lung transplant in all patients and for those with pulmonary vascular resistance (PVR) ≥6 Wood units.

The median waitlist time for all double lung transplants started at 378.5 days in 2003, with a drop to 86 days by 2006 and now down to 35 days in 2022. A similar trend was seen in patients with high PVR started with a median waitlist time of 339 days in 2003 to 54 days in 2006 and 24 days in 2022 (Figure 4).

## Comment

The landscape of lung transplantation with high PVR has evolved significantly from 2003 to 2022. A notable finding in this analysis is a linear increase in mortality risk for patients with PVR ≥2 Wood units, intensifying significantly beyond 6 Wood units, underscoring the need for prompt and careful management in such patients. Moreover, the correlation between higher transplant center volumes and superior patient outcomes has become increasingly evident. Centers performing a greater number of lung transplants annually benefit from their experience, higher procedural volumes, and access to advanced care technologies, all of which contribute to better patient survival rates.

There has also been a reduction in waitlist times and greater use of ECMO during transplantation. Although there was no difference in ECMO use at listing, there was an increased use of ECMO in patients with an elevated PVR during transplantation. This is consistent with prior work that showed elevated rates of ECMO use intraoperatively in patients with PH with elevated PVR due to pulmonary fibrosis and more complicated postoperative course in that subset.^6^ This highlights the importance of early identification of this higher risk population of patients.

Specifically in PAH management, lung transplantation trends are crucial. Whereas PAH remains incurable, the last 2 decades have seen significant advances. Improved understanding of PAH pathophysiology has revealed new therapeutic targets in prostaglandin, nitric oxide, and endothelin pathways. For chronic thromboembolic PH, treatments now include pulmonary endarterectomy and pulmonary artery balloon angioplasty, expanding options for patients.^7^ However, lung transplantation still stands as the ultimate treatment for cases refractory to medical therapy, with evolving practices in recipient selection and surgical techniques, including the use of mechanical circulatory support.

Given the unpredictable progression of PAH, early referral to a transplant center is crucial. This allows the timely identification and management of risk factors, understanding that some patients may not be viable candidates for transplantation because of various contraindications. However, surgical and medical care advancements have rendered some previous contraindications obsolete.^8^ Regular follow-ups for patients on the transplant waiting list are essential, particularly for PH patients, as any clinical status change may necessitate expedited transplantation.^9^^,^^10^ Participation in clinical trials at tertiary referral centers offers additional opportunities for patients with idiopathic PAH.

Across surgical subspecialties, there is a consistent association between institutional experience with surgical procedures and improved clinical outcomes. This is particularly relevant for solid organ transplantation, for which the scarcity of donor organs necessitates effective resource utilization. The relationship between surgical volume and outcomes is an essential consideration in lung transplantation, as evidenced by the findings from Yang and coworkers.^5^

### Limitations

This study has several limitations. First, the data were obtained from the United Network for Organ Sharing registry, which is retrospective and observational in nature, potentially introducing selection bias. Second, detailed information on cardiopulmonary bypass use during lung transplantation is not available in the data set, limiting our ability to analyze its impact alongside ECMO. Last, the generalizability of our findings to nontransplant centers or international practices may be limited, given the focus on US data.

### Conclusion

The past 2 decades have marked a period of continuous evolution and improvement in the management of PAH, particularly regarding lung transplantation. The growth in lung transplants performed globally reflects not only these advancements but also the ongoing need for innovation, research, and collaborative efforts to further improve patient outcomes in this challenging clinical domain.
