# Supplementary material for: Lung Transplantation With Elevated Pulmonary Vascular Resistance: Insights From the United Network for Organ Sharing Database
Source: Ann Thorac Surg Short Rep. 2025 Feb 25;3(3):784–90. doi: 10.1016/j.atssr.2025.01.022 (PMC12559271; doi:10.1016/j.atssr.2025.01.022)
Supplement: Supplementary Figure 1 and Supplementary Tables 1-2 [file mmc1.docx]

**Supplemental Figure 1:**


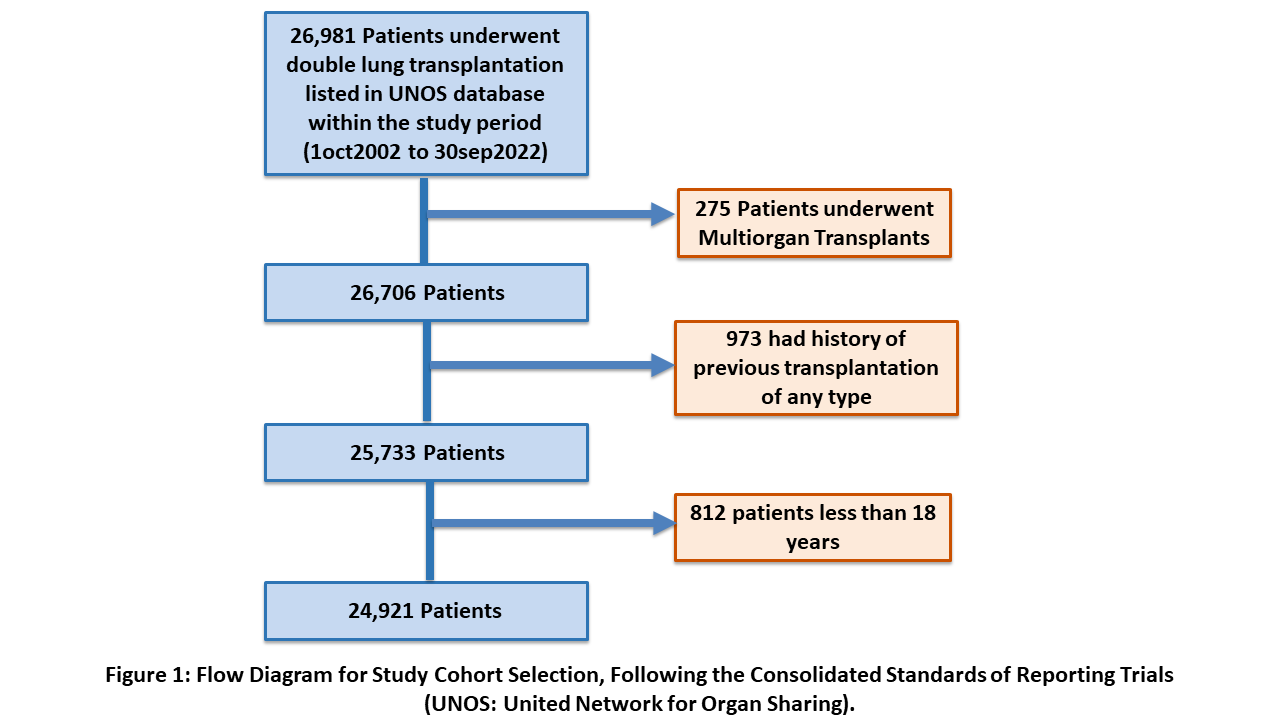


**Supplemental Table 1:** **Patient demographics and clinical characteristics: overall double lung transplant Cohort Versus High-PVR Group (PVR> 6)**

|  | All patients undergoing lung transplantation.  (n= 24,921) | High-PVR (>6 WU) Subset  (n=2,755) | p-Value |
| --- | --- | --- | --- |
| Recipient Female,n(%) | 10463(42%) | 1504(54.6%) | <0.05 |
| Recipient Ethnicity,n(%) |  |  | <0.05 |
| White,n(%) | 19692(79%) | 1847(67%) |  |
| Black,n(%) | 2510(10.1%) | 492(17.9%) |  |
| Hispanic/Latino,n(%) | 2005(8%) | 285(10.3%) |  |
| Others,n(%) | 714(2.8%) | 131(4.7%) |  |
| Recipient Age, [median(IQR)] years | 57(IQR:47-64) | 56(IQR:46-63) | 0.048 |
| Recipient Diabetes,n(%) | 4720(18.9%) | 462(16.7%) | <0.05 |
| Recipient History of Cerebrovascular Disease,n(%) | 21(0.8%) | 7(3.1%) | <0.05 |
| Recipient History of Malignancy,n(%) | 1883(7.6%) | 172(6.2%) | <0.05 |
| Recipient History of Cigarette Use,n(%) | 12990(55.3%) | 1354(51.1%) | <0.05 |
| Recipient on Ventilator at Listing,n(%) | 811(3.3%) | 66(2.4%) | <0.05 |
| Recipient on Ventilator during transplantation,n(%) | 1750(7%) | 167(6.1%) | <0.05 |
| Recipient on ECMO at Listing,n(%) | 673(2.7%) | 68(2.5%) | 0.425 |
| Recipient on ECMO during transplantation,n(%) | 1438(5.8%) | 208(7.5%) | <0.05 |
| Medical acuity |  |  | <0.05 |
| In ICU,n(%) | 3476(13.9%) | 554(20.1%) |  |
| Hospitalized but not in ICU,n(%) | 2481(10%) | 365(13.2%) |  |
| Not Hospitalized,n(%) | 18962(76.1%) | 1836(66.6%) |  |
| Donar Age, [median(IQR)] years | 33(IQR:23-46) | 34(IQR:23-47) | <0.05 |
| Recipient Body Mass Index (BMI), [median(IQR)]kg/m2 | 25.2(IQR:21.5-28.6) | 25.1(IQR:21.7-28.5) | 0.168 |
| Recipient Creatinine, [median(IQR)]mg/dl | 0.8(IQR:0.7-1) | 0.9(IQR:0.7-1.1) | <0.05 |
| Lung Allocation Score, [median(IQR)] | 37.6(IQR:33.7-45.4) | 43.6(IQR:37.3-55.5) | <0.05 |
| Recipient Pulmonary Artery Systolic Blood, [median(IQR)] mm/Hg | 40(IQR:32-50) | 73(IQR:60-87) | <0.05 |
| Recipient Pulmonary Diastolic Pressure, [median(IQR)] mm/Hg | 17(IQR:12-23) | 30(IQR:25-38) | <0.05 |
| Recipient Mean Pulmonary Pressure, [median(IQR)] mm/Hg | 26(IQR:21-33) | 47(IQR:39-55) | <0.05 |
| Recipient Pulmonary Capillary Wedge Pressure, [median(IQR)] mm/Hg | 10(IQR:7-14) | 10(IQR:7-13) | <0.05 |
| Recipient Pulmonary vascular resistance, [median(IQR)] WU | 2.8(IQR:1.9-4.2) | 8.3(IQR:6.8-11.2) | <0.05 |
| Recipient Cardiac Output, [median(IQR)] L/min | 5.2(IQR:4.4-6.2) | 4.1(IQR:3.3-4.8) | <0.05 |
| Recipient Forced expiratory volume (FEV1), % predicted | 46(IQR:36-60) | 56(IQR:41-73) | <0.05 |
| Recipient Forced vital capacity (FVC), % predicted | 34(IQR:21-53) | 51(IQR:35-67) | <0.05 |
| Recipient Oxygen requirement, [median(IQR)] L/min | 3(IQR:2-5) | 4(IQR:2-6) | <0.05 |
| Ischemic Time, [median(IQR)], hours | 5.6 (IQR:4.7-6.7) | 5.6(IQR:4.7-6.8) | 0.299 |
| Top 5 primary diagnosis (Recipient) | 1. Idiopathic Pulmonary Fibrosis (29.5%) 2. COPD/Emphysema (21.8) 3. Cystic Fibrosis (13.7%) 4. Secondary Pulmonary Fibrosis (6.3%) 5. Sarcoidosis (3.5%) | 1. Idiopathic Pulmonary Fibrosis (27.5%) 2. Pulmonary Arterial Hypertension (20.5%) 3. Sarcoidosis(8.4%) 4. COPD/Emphysema(8.1%) 5. Secondary Pulmonary Fibrosis (7.3%) |  |

**Legend**: PVR-Pulmonary vascular resistance, ICU- Intensive Care Unit, IQR- Interquartile Range, kg/m2-Kilogram per square meter, mm/Hg-millimeters of mercury, L/min-Liter Per Minute.

**Supplemental Table 2:** **Top causes of respiratory failure and one year mortality**

|  |  | **One year mortality** | |
| --- | --- | --- | --- |
|  | **Top Causes of Respiratory Failure** | **High PVR group** | **low PVR group** |
| **1** | **Idiopathic Pulmonary Fibrosis (N=7,381)** | 14.5% (111/765) | 12.9% (851/6616) |
| **2** | **COPD/Emphysema (N=5,233)** | 16.5% (30/182) | 10.4% (526/5051) |
| **3** | **Cystic Fibrosis (N=3,438)** | 8.8% (10/114) | 10.1% (335/3324) |
| **4** | **Pulmonary Fibrosis Unspecified (N=1,795)** | 15.1% (38/252) | 15.1% (233/1543) |
| **5** | **Sarcoidosis (N=852)** | 15.8% (35/222) | 15.4% (97/630) |
| **6** | **Pulmonary Arterial Hypertension (N=849)** | 15% (86/574) | 17.4% (48/275) |
|  | **Total** | 15% (310/2109) | 12% (2090/17439) |

**Legend**: PVR-Pulmonary vascular resistance
